# A Plant Stress-Responsive Bioreporter Coupled With Transcriptomic Analysis Allows Rapid Screening for Biocontrols of Necrotrophic Fungal Pathogens

**DOI:** 10.3389/fmolb.2021.708530

**Published:** 2021-09-03

**Authors:** Katharina Belt, Rhonda C. Foley, Cathryn A. O’Sullivan, Margaret M. Roper, Karam B. Singh, Louise F. Thatcher

**Affiliations:** ^1^ Commonwealth Scientific and Industrial Research Organisation (CSIRO) Agriculture and Food, Floreat, WA, Australia; ^2^ Commonwealth Scientific and Industrial Research Organisation (CSIRO) Agriculture and Food, St Lucia, QLD, Australia; ^3^ Commonwealth Scientific and Industrial Research Organisation (CSIRO) Agriculture and Food, Acton, ACT, Australia

**Keywords:** GSTF7, glutathione S-transferase, *Rhizoctonia solani*, *Sclerotinia sclerotiorum*, plant defense signaling, bacteria–plant interaction

## Abstract

*Streptomyces* are soil-borne Actinobacteria known to produce a wide range of enzymes, phytohormones, and metabolites including antifungal compounds, making these microbes fitting for use as biocontrol agents in agriculture. In this study, a plant reporter gene construct comprising the biotic stress-responsive glutathione S-transferase promoter GSTF7 linked to a luciferase output (GSTF7:luc) was used to screen a collection of Actinobacteria candidates for manipulation of plant biotic stress responses and their potential as biocontrol agents. We identified a *Streptomyces* isolate (KB001) as a strong candidate and demonstrated successful protection against two necrotrophic fungal pathogens, *Sclerotinia sclerotiorum* and *Rhizoctonia solani*, but not against a bacterial pathogen (*Pseudomonas syringe*). Treatment of Arabidopsis plants with either KB001 microbial culture or its secreted compounds induced a range of stress and defense response-related genes like pathogenesis-related (PR) and hormone signaling pathways. Global transcriptomic analysis showed that both treatments shared highly induced expression of reactive oxygen species and auxin signaling pathways at 6 and 24 h posttreatment, while some other responses were treatment specific. This study demonstrates that GSTF7 is a suitable marker for the rapid and preliminary screening of beneficial bacteria and selection of candidates with potential for application as biocontrols in agriculture, including the *Streptomyces* KB001 that was characterized here, and could provide protection against necrotrophic fungal pathogens.

## Introduction

Pathogens and pests cause severe damage to crops and are estimated to cost the global economy billions of dollars each year (Bradshaw et al., 2016; Savary et al., 2019). Yield reductions, on average, range from 20 to 40%, or higher (Ficke et al., 2017; Willocquet et al., 2018). Some disease management strategies using chemical fungicides are losing effectiveness as pathogens evolve resistance and overcome their modes of action (Hahn 2014). Furthermore, the increasing use of chemicals has concerning impacts on the environment and human health (Nicolopoulou-Stamati et al., 2016). New environmentally friendly strategies with alternate modes of action are needed to provide growers with sustainable disease control tools.

Beneficial plant-interacting microbes offer new possibilities to protect the farming industry from pathogens. Many of these microbes create symbiotic relationships with plants and promote plant growth and development, nutrient uptake, and tolerance to abiotic and biotic stresses (Rey and Dumas 2017; Woo and Pepe, 2018; Reis et al., 2020; Romano et al., 2020). These microbe–plant associations play major roles in the ecosystem and can take place at the root level or the shoot level, also known as the rhizosphere (in soil) and the phyllosphere (plant aerial parts), respectively. Within this environment, microbial communities interact within themselves but also with their host plants, which, in a beneficial relationship, can improve plant fitness to environmental constraints (Schulz and Boyle 2005; Hardoim et al., 2015; Govindasamy et al., 2018). By producing antimicrobials or influencing the host’s biosynthesis of primary and specialized metabolites, enhanced root and shoot growth and increased plant resistance against pathogens have been reported (Dias et al., 2009; Chamam et al., 2013; Finkel et al., 2017; Porter et al., 2020). Still, we are just at the beginning of understanding how these microbes interact with their host plant and what factors are needed to trigger production of specialized metabolites (Maheshwari 2012; Etalo et al., 2018). A major challenge in the application of biocontrol is to translate a well-performing microbe under laboratory conditions to the field (Constantin et al., 2020). The effectiveness of a biocontrol can differ between laboratory and field conditions due to landscape composition and interactions with other organisms (Perez-Alvarezet al., 2019).

Actinobacteria represent a diverse group within the phyla of bacteria and play important ecological roles in the soil, rhizosphere, and phyllosphere (Zhang et al., 2019). Many Actinobacteria are known to protect plants against pathogens and promote plant growth, with a small proportion shown to be pathogenic to specific plant hosts (Ventura et al., 2007; Sathya et al., 2017; Worsley et al., 2020). These microbes vary highly in their morphology, life style, and specialized metabolite production (Hardoim 2018). At least 3,000 potential compounds produced by Actinobacteria have been reported to have pesticide, herbicide, or plant growth activity (Sathya et al., 2017). However, genome mining studies have shown that these microbes have the potential of making thousands of bioactive compounds, leaving an extensive opportunity for new natural compound discoveries to benefit agriculture and industry (Sathya et al., 2017).

The mechanisms behind the beneficial interaction of Actinobacteria and their host plant that leads to increased plant fitness and protection from biotic stress are poorly understood. Plant defense responses rely on recognition of molecular patterns exposed by pathogens (PAMPs) such as flagellin or peptidoglycans that trigger an immune response in the plant (Macho and Zipfel 2014; Stringlis et al., 2018). Subsequent activation of defense responses depends on signaling molecules and plant hormones such as hydrogen peroxide (H_2_O_2_), salicylic acid (SA), jasmonic acid (JA), and ethylene (Pieterse et al., 2014). Dynamic signaling processes activated by these molecules induce a range of stress markers that initiate plant defense in either a general or specific stress response. A group of ubiquitous and multifunctional enzymes with high inducibility and involvement in biotic and abiotic stress responses are the plant glutathione S-transferases (GSTs) (Marrs 1996; Edwards et al., 2000; Gullner et al., 2018). Early expression of GSTs is used as a marker of defense responses against pathogen attack (Asano et al., 2012; Thatcher et al., 2019). Elevated gene expression or protein accumulation of several GSTs was observed in plants treated with beneficial microbes that have biocontrol properties or the ability to activate induced systemic resistance (ISR), a process whereby beneficial microbes prime the plant for enhanced defense against pathogens (Pieterse et al., 2014; Gullner et al., 2018). The exact role of GSTs during interactions with beneficial microbes is yet to be elucidated, but it is thought that they have roles as antioxidants such as reactive oxygen species (ROS) scavenging or in the detoxification of pathogen toxins (Gullner et al., 2018). Both would have important implications in reducing the effects of necrotrophic pathogens that destroy host plant tissues through secreted toxins and ROS-induced damage (Laluk and Mengiste 2010; Mengiste 2012; Foley et al., 2016).

The Arabidopsis GST Phi 7 (GSTF7) gene is strongly induced after treatment with the ROS molecule hydrogen peroxide (H_2_O_2_) (Sappl et al., 2009) and is expressed in almost all plant tissues (http://bar.utoronto.ca/; Supplementary Figure S1). GSTF7 expression or protein abundance is strongly increased following infection by foliar- or root-infecting necrotrophic fungal pathogens including *Sclerotinia sclerotiorum, Alternaria brassicicola*, and *Fusarium oxysporum* (Garg and Smith 2015; Seifbarghi et al., 2017; Thatcher et al., 2018; Zhou et al., 2019). GSTF7 expression is also highly induced in Arabidopsis plants treated with the plant defense inducing Actinobacteria biocontrol strain *Streptomyces* AgN23 or its secreted extracellular compounds (Vergnes et al., 2019). These properties suggest that GSTF7 is an ideal marker gene candidate for screening of beneficial microbial strains for potential to activate or manipulate plant defense.

The aim of this research was, first, to design a GSTF7:luciferase (GSTF7:luc) reporter system in stably transformed Arabidopsis for non-destructive observation of GSTF7 expression *in planta* and in real time; second, to develop a high-throughput bioluminescence assay to screen large collections of microbes for their ability to manipulate GSTF7 expression and narrow the number of candidates for detailed downstream functional testing for biocontrol or ISR properties; and third, to test the utility and validity of this approach against a unique collection of Actinobacteria and identify strains that induce GSTF7 and might be used as biocontrols. One standout strain, *Streptomyces* KB001, was selected for detailed follow-up studies. Global transcriptome analysis of KB001-inoculated plants and *in planta* disease protection assays demonstrated that KB001 and its secreted antifungal compounds and induced plant defense responses were contributing to protection of Arabidopsis plants against two necrotrophic fungal pathogens.

We demonstrate that our GSTF7 reporter system can be used as a high-throughput tool for rapid microbial screening and selection of biocontrol and ISR candidates for potential agricultural applications.

## Materials and Methods

### Actinobacteria Growth and Preparation of Actinobacteria Spore Suspensions for Plant Inoculations

Actinobacteria strains were sourced from two collections; one developed based on selection of strains with biocontrol potential (Roper et al., 2016; O’ Sullivan et al., 2021) and another based on potential for biosurfactant and soil wettability properties (Roper 2004). Strain KB001 was isolated from wheat plants as described previously (Roper, 2004; Roper et al., 2016) and was identified as *Streptomyces tendae* based on genome sequencing (16S rRNA sequence available under GenBank accession number MZ669887). Strains were grown on plates containing half-strength potato dextrose agar (PDA) at 26°C in the dark. Once the Actinobacteria started to sporulate (1–2 weeks), spores were gently scraped off the plates in sterile water and filtered through miracloth to remove mycelial fragments. The concentration of spore stocks was calculated from a dilution series on PDA plates ranging from 10^1^ to 10^8^. An end concentration of 10^6^ cfu/ml was prepared in sterile water for use in all assays.

### Design of GSTF7:luc Arabidopsis Plants

The GSTF7:luc transgenic plants containing the GSTF7 promoter (∼1,500 bp) fused to the LUC + gene (Promega, Madison, WI, United States) was made using a binary vector [pYRO (Thatcher et al., 2007)] with a promoterless LUC + coding fragment containing BamHI and HindIII restriction sites for promoter cloning. The GSTF7 (At1g02920) promoter fragments were amplified by PCR using the primer pairs 5′-GCG​GGA​TCC​AAC​TAC​CAA​TTA​ATG​TTG​AT-3′ and 5′-GGG​GAA​GCT​TGT​TAA​GAC​TT TGTTCTTA-3′, which included added restriction sites for cloning and used genomic DNA from the Col-0 ecotype of Arabidopsis as a template. The construct was sequenced, moved into *Agrobacterium* AGL1, and transformed into the Col-0 ecotype. Homozygous T3 lines with one insert were screened for uniform luciferase activity, and one was selected for subsequent experiments.

### Growth of Arabidopsis for H_2_O_2_ Bioluminescence Time Course

GSTF7:luc plants were grown in the presence of Actinobacteria strains sourced from two unique collections (O'Sullivan et al., 2021; Roper 2004; Roper et al., 2016) to identify strains that could induce or enhance H_2_O_2_-dependent plant responses. As a positive control, H_2_O_2_ treatment was also included, and a water-only treatment was used as a negative control. Initially, 40 strains were screened to confirm that Actinobacteria could indeed induce GSTF7:luc activity. A total of twelve of the most promising candidates were selected for this study (Supplementary Table S1). On measuring GSTF7:luc luminescence following bacterial inoculation onto GSTF7:luc plants, bacterial strains involved in plant stress signaling should show a higher luminescence signal. Arabidopsis seedlings were incubated with spores from each of the 12 Actinobacteria strains separately, and luciferase activity was measured over 2 days. The dataset was transformed to fit a logarithmic scale to the base of 2 (log2), which was suitable for statistical analysis (Supplementary Figure S2). Agar plates contained 1x Murashige and Skoog (MS) salts (Gibco; 4.3 g/L) and 0.8% agar, with 3% sucrose, pH adjusted to 5.7 with 1 M KOH. Plates for the luciferase assay were supplemented with 50 µM luciferin (Biosynth AG), added after autoclaving the medium. Arabidopsis GSTF7:luc seeds were surface-sterilized for 15 min with 70% ethanol, washed twice in water, and incubated for 2 days at 4°C for vernalization. Twenty seeds were plated onto round 55-mm MS plates containing the luciferin and incubated vertically in the growth room (22°C, 16-h light:8-h dark photoperiod). Next, 3 ml of 1 mM H_2_0_2_ treatment was pipetted onto the agar plates containing 4-day-old seedlings. The solution was drained off after 40 min. The plates were left in the dark chamber in an EG & G Berthold Molecular Light Imager (Berthold NightOWL) during the time course experiments, with measurements taken every 60 min.

### GSTF7:luc Reporter Bioluminescence Assay to Screen Actinobacteria

To first test the validity of Actinobacteria inducing GSTF7:luc activity, GSTF7:luc seeds were sterilized and plated out on MS luciferin plates and grown upright as described above. Next, 3 ml of Actinobacteria spores (10^6^ cfu/ml) was added to the plate, and luminescence was measured hourly over 3 days. Bioluminescence was captured using a Nightshade Molecular Light Imager (Berthold Technologies) with 10 s of exposure time. Bioluminescence was quantified using IndiGo (v2.0.3.0) software (Berthold Technologies). A total of 40 isolates of Actinobacteria were screened this way, and 12 isolates that showed high luminescence responses were chosen for this study (Supplementary Table S1). For development of the high-throughput assay in 24-well format, GSTF7:luc seeds were sterilized and plated onto MS media in the absence of luciferin and grown for 5 days in a growth room at 22°C and in a 16-h light/8-h dark cycle. Then, 5-day-old seedlings were removed from the MS plates and transferred to 24-well plates containing 1x MS media and 50 µM luciferin. Each of the 24 wells contained one seedling. Plants were incubated in the growth room for 2 days, followed by the addition of 100 µl of Actinobacteria spore solution (10^6^ cfu/ml) to each well. Once Actinobacteria were added to the 24-well plates, bioluminescence was measured using the Nightshade Molecular Light Imager every 60 min over 3 days. Actinobacteria strains that showed significant GSTF7:luc induction over a water control treatment were selected for further assays. The plates were left in the light imager for the duration of the time course.

### Quantitative Real-Time (RT)-PCR in GSTF7:luc Arabidopsis Plants

A subset of selected Actinobacteria strains were tested to determine if these strains would enhance plant stress responses and provide protection against *S. sclerotiorum* infection, a representative, necrotrophic fungal pathogen with a broad host range. Two-week-old Arabidopsis plants were grown in MS media in 24-well plates. Plants were incubated with 100 µl of Actinobacteria spore suspension and incubated for 3 days in the growth room (22°C and 16-h light/8-h dark cycle). During this time, Actinobacteria were expected to colonize the plant and activate signaling pathways and subsequent downstream defenses, which were predicted to provide some level of protection from disease. Three days after application of the Actinobacteria treatments, plants were infected with *S. sclerotiorum*, and the expression of GSTF7 and representative defense marker genes was measured through qPCR at 5 and 48 h post-infection. *S. sclerotiorum* mycelium fragments (∼10^6^ fragments/ml) were prepared as described elsewhere (Thatcher et al., 2017), and 5 µl was pipetted onto the rosettes of each seedling in the 24-well plate. Controls without Actinobacteria were measured as well (H_2_O control). To gain enough plant material for analysis, tissue for one replicate was pooled from four seedlings and then frozen in liquid nitrogen and stored at −80°C. Four biological replicates for each treatment and control (H_2_O) were collected in total. Whole-plant RNA was isolated using a total RNA kit (Sigma, United States), followed by DNase treatment using TURBO DNase (Ambion). Total RNA (1 µg) was used to synthesize first-strand cDNA using a SuperScript III RT reagent kit (Invitrogen, United States). Quantitative RT-PCR was carried out using 2x SYBR green Mastermix (Biorad, United States). The list of primers can be found in Supplementary Table S2. Arabidopsis ubiquitin-conjugating enzyme 9 (UBC9) was used as the reference gene as its expression was found to be stable and suitable for normalization in Arabidopsis (Czechowski et al., 2005). The qRT-PCR analysis was performed using the CFX384 Real-Time System (Biorad). The program can be found in Supplementary Table S3. The cDNA quantities of target genes were calculated using CFX Manager (version 3.1) (Biorad).

For the KB001 filtrate (KB001_F) vs. microbe (KB001_M) culture qRT-PCR assay, 100 µl of KB001_M or KB001_F was used. A total of 100 µl of KB001 spores were grown in 100 ml of CYPS media for 2–3 weeks and shaken at 26°C. Then, 100 µl of KB001_M or KB001_F was added to 2-week-old plants, and expression of genes was measured 6 and 24 h posttreatment. KB001_F samples were processed by filtering the KB001_M samples and spent media through a 0.22-µm filter to remove mycelia and spores.

### *Sclerotinia* Disease Assay on Arabidopsis Plants

Arabidopsis Col-0 was grown in Arabidopsis soil mix (containing dolomite, agricultural lime, perlite, cocopeat, and vermiculite) for 4 weeks in a growth room (22°C and 16-h light/8-h dark cycle). Actinobacteria were grown for 2–3 weeks in liquid CYPS media (3 g yeast extract and 5 g casein in 1 L) at 26 °C and shaken at 200 rpm. Previous studies identified CYPS as a suitable culture media to induce compound production in Actinobacteria (Pan et al., 2019). Plants were sprayed with Actinobacteria microbial culture media (24 h and 3 days prior to *Sclerotinia* infection) or Actinobacteria culture filtrate 24 h prior to *Sclerotinia* infection. Whatman paper discs were soaked in *Sclerotinia* mycelia and transferred onto 4–week-old Arabidopsis leaves (3 per plant). Plastic domes were placed over inoculated plants to maintain humidity. Severity of disease symptoms was monitored daily, and length of necrotic lesions was calculated 3 and 4 days post-infection using ImageJ software.

### *Rhizoctonia* AG-2 Disease Assay in Arabidopsis

Arabidopsis Col-0 plants were grown and treated with Actinobacteria isolate KB001 as described above. Millet seeds infected with the *Rhizoctonia solani* strain *AG2-1* [ZG5: WAC9767 (Foley et al., 2013)] were cultured on water agar media. Small round agar plugs (0.5 cm in diameter) of developed mycelia were transferred onto 4-week-old Arabidopsis leaves for infection. *R. solani* is a root-infecting pathogen; however, studies showed that it can infect Arabidopsis leaf tissue (Kidd et al., 2021). To test whether KB001 would protect Arabidopsis leaf tissue from *R. solani* infection, similar to *S. sclerotinia*, we chose this method. Plastic domes were placed over inoculated plants to maintain humidity. Severity of disease was captured in images, and lesions were analysed using ImageJ software 3 and 4 days post-infection.

### Testing KB001 for Antifungals Against *Rhizoctonia* and *Sclerotinia*

Actinobacteria isolate KB001 was grown on half-strength PDA media for 2 weeks as described above. KB001 was challenged with *R. solani* and *S. sclerotiorum* as described in the study by O’Sullivan et al., 2021. In brief, agar plugs of *R. solani* and *S. sclerotiorum* were added opposite to KB001 on the PDA plate. Control PDA plates with fungi only were prepared as a reference. Inhibition zones between KB001 and the fungus was captured by photographs after 1 week.

### *Pseudomonas* Disease Assay in Arabidopsis

Arabidopsis Col-0 plants were grown and treated with KB001 as described above. *Pseudomonas syringae pv* tomato DC3000 grown on plates were spread over a fresh King’s B medium plate (20 g Bacto-Proteose peptone, 10 ml glycerol, 1.5 g K_2_HPO_4_, and 1.5 g MgSO_4_, pH 7.2, in 1 L) and grown for 24 h to obtain a lawn. Bacteria were resuspended in 10 ml of 10 mM MgCl_2_ that was added to the plate and shaken for 10 min. Bacteria were diluted to an OD_600_ (Katagiri et al., 2002) of 0.05 (∼25 million CFU/ml). Plants (4 weeks old) were sprayed with *Pseudomonas* suspension, and development of disease symptoms was observed after 2 weeks.

### RNAseq Analysis of Arabidopsis Plants Treated With Actinobacteria

RNA samples used for qRT-PCR assays were also used for RNAseq experiments. A total of four biological replicates for each KB001 treatment group [3-week-old CYPS KB001 microbe (KB001_M) culture or filtrate (KB001_F)] and the nontreated control were prepared. RNAseq TruSeq libraries were generated from 1 µg of total RNA, and sequencing was performed by the AGRF on an Illumina Novaseq S4 Lane using a paired-end read length of 150 bp. The quality control of samples was performed using FastQC (Zhou et al., 2018). Paired-end reads were matched to the Arabidopsis TAIR10 genome reference using kallisto (Bray et al., 2016). Differential gene expression analysis was performed in R using the DeSeq2 package, version 4.0.2 (Varet et al., 2016). A list of highly and lowly expressed genes based on log2 fold change and a *p*-value smaller than 0.05 (adjustment using Benjamini–Hochberg correction) between the nontreated control (NTC) and the KB001 treatments at the timepoints 6 and 24 h can be found in supplemental data (Supplementary Table S4). Gene annotations and descriptions were sourced from the TAIR10 database. The raw data files used for this analysis can be found on NCBI under the Bio project ID PRJNA752867 (www.ncbi.nlm.nih.gov/bioproject/752867).

## Results

### GSTF7:luc Construct Can Be Used as a Selecion Marker to Screen Actinobacteria With Biocontrol Potential

**FIGURE 1:**
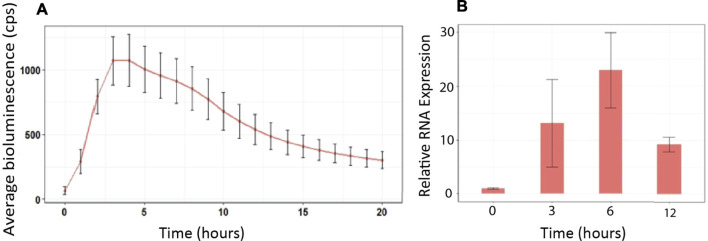
GSTF7:luc expression mimics endogenous GSTF7 expression following H_2_O_2_ treatment. **(A)** Four-day-old GSTF7:luc seedlings were treated with 1 mM H_2_O_2_ for 40 min, and the bioluminescence was measured over 20 h using a CCD camera. The average light units from 20 seedlings at each hour, with standard errors, are presented. **(B)** RNA samples were extracted before treatment (time = 0), 3, 6, and 12 h following 1 mM H_2_O_2_ treatments. Expression levels of GSTF7 were determined by qPCR. Illustrated are normalized expression levels to cyclophilin (At2g29960). Average was calculated out of 3 biological replicates.

A rapid *in vivo* screening method to observe defense gene activation in real time following microbial inoculation and manipulation of ROS signaling was developed. First, we generated transgenic Arabidopsis plants expressing the luciferase reporter gene under the control of the GSTF7 promoter. To confirm that the luciferase reporter construct faithfully mimicked endogenous GSTF7 expression, we treated GSTF7:luc plants with the ROS molecule hydrogen peroxide (H_2_O_2_), known to strongly induce GSTF7 expression (Sappl et al., 2009). GSTF7:luc expression peaked between 3 and 6 h post H_2_O_2_ treatment (Figure 1A), which reflected the expression profile of the endogenous GSTF7 gene as determined by qPCR (Figure 1B).

**FIGURE 2:**
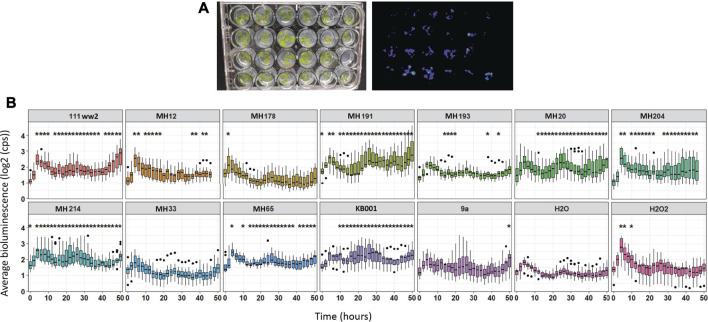
Screening of Actinobacteria to identify candidates with potential for biocontrol. Arabidopsis seedlings were grown on MS media in 24-well plates for 10 days. Spores of Actinobacteria were added around the roots of seedlings, and GSTF7:luc luminescence was measured hourly over 50 h. Water and 1 mM H_2_O_2_ treatments were added to plants as references. Average was calculated out of 12 biological replicates. Dots refer to outlier samples. A *t*-test was performed to determine significant differences between samples compared to water treatment. *p < 0.05.

Of the 12 Actinobacteria strains tested, six showed significantly higher (*p* < 0.05) GSTF7:luc activity (Figure 2) over the majority of the time course compared to GSTF7:luc plants treated with mock (water). Mock treatment also showed a slight increase in activity over the first 5 h. Plants are reactive to touch, which might explain this short increase in luciferase activity. For further analysis, two high luminescence and two lower luminescence strains were selected. The strains MH191 and KB001 showed high activity over the majority of the 50-h time course and showed similar high luminescence values to those of the H_2_O_2_ control treatment for activation of GSTF7:luc expression. In contrast, strains such as MH33 and 9a showed mild differences from the mock. Based on these results, strains KB001 and MH191 were selected as positive biocontrol candidates and MH33 and 9a as weaker strains for follow-up experiments.

### Pretreatment With Actinobacteria Strains MH191 and KB001 Enhances Plant Defense Gene Expression Against the Necrotrophic Pathogen *Sclerotinia sclerotiorum*

Treatment of MH191 and KB001 on Arabidopsis was tested to determine if these strains would enhance plant stress responses and provide protection against *S. sclerotiorum*. Arabidopsis seedlings were incubated for 3 days with MH191 and KB001 as well as MH33 and 9a for reference. The expression of GSTF7 and representative defense marker genes were measured through qPCR at 5 and 48 h post *Sclerotinia* infection. This would be representative of early and later activation of defense markers in Arabidopsis (Schenk et al., 2000; Dos Santos et al., 2003).

**FIGURE 3:**
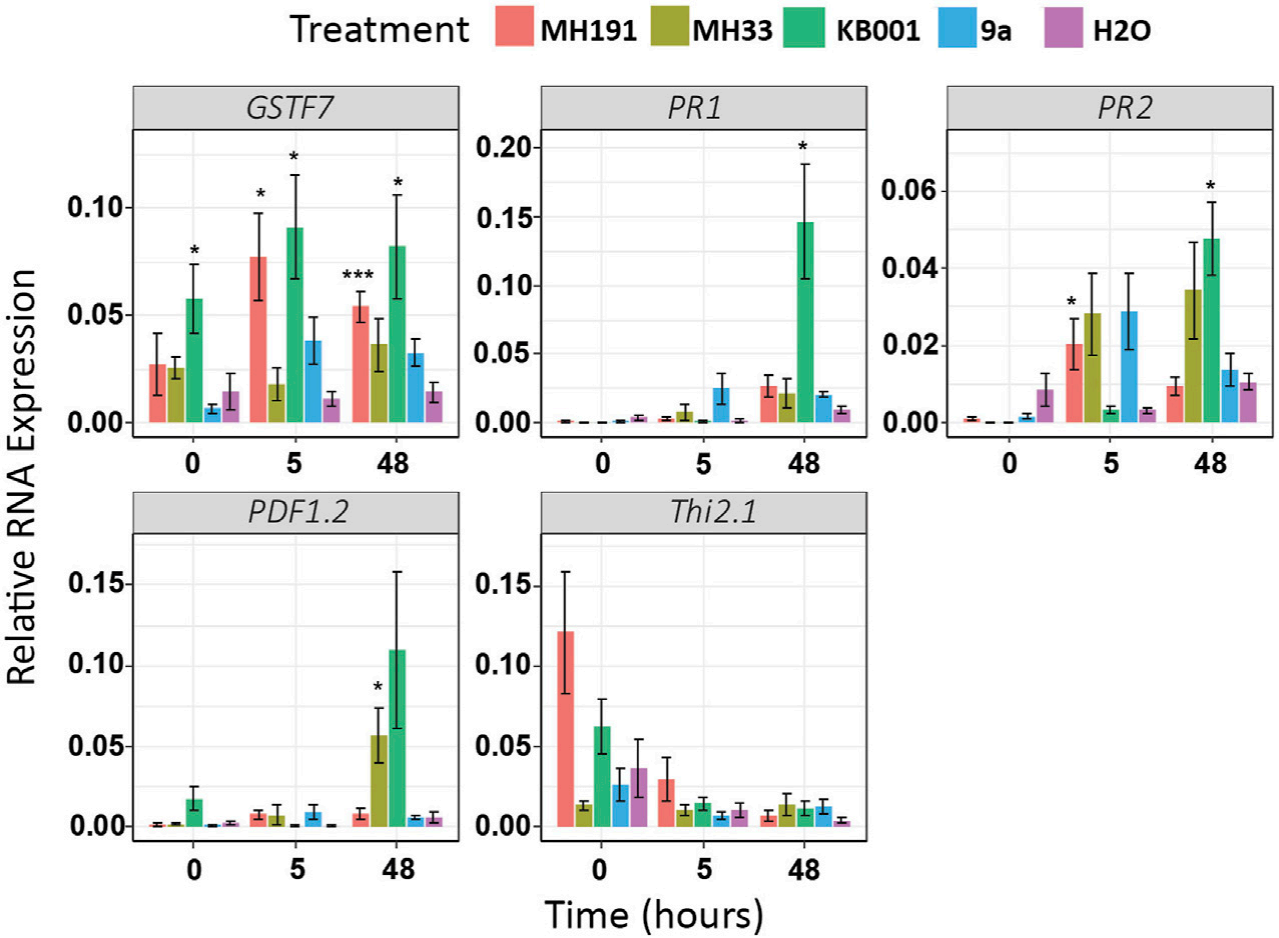
Gene expression of stress markers increased in Actinobacteria-treated Arabidopsis plants. Expression levels of SA (PR1 and PR2) and JA (PDF1.2 and Thi2.1) induced stress markers were determined using qRT-PCR. Samples were extracted 3 days after treatment with Actinobacteria but before infection (time = 0), 5 and 48 h post *Sclerotinia* infection. Expression values were averaged, and 6–8 biological replicates were normalized to UBC. A *t*-test was used to determine significant differences to water treatments. *p < 0.05; **p < 0.01; ***p < 0.001.

As illustrated in Figure 3, GSTF7 expression was strongly induced in plants pretreated with the Actinobacteria strains MH191 or KB001. Several other stress markers were also significantly induced in these plants. We tested ethylene-, jasmonic acid (JA)-, and SA-dependent stress markers. In particular, KB001 showed the highest expression across most genes tested, especially at 48 h post *Sclerotinia* infection, making it the strongest candidate for further analysis. This included both SA (PR1 and PR2) and JA marker genes (PDF1.2), indicating a broad influence on plant hormone signaling pathways. Plants treated with MH191 showed a strong JA response (Thi2.1), while strains MH33 or 9a showed a more variable or lower defense gene expression response.

Overall, these results indicate that plants colonized with Actinobacteria strains MH191 and KB001 identified through our GSTF7:luc screen showed higher expression of endogenous GSTF7 and of stress and defense marker genes than those given the mock treatment. KB001 stood out as it showed the highest response across all marker genes tested. The isolate was identified morphologically as a *Streptomyces* (Roper 2004) and was confirmed by 16s rDNA sequencing as a species of the *Streptomyces* genus (O'Sullivan et al., 2021). No visible differences in Arabidopsis plants could be observed at this point.

### Actinobacteria Culture Filtrates Enhance Stress Marker Expression in Arabidopsis

**FIGURE 4:**
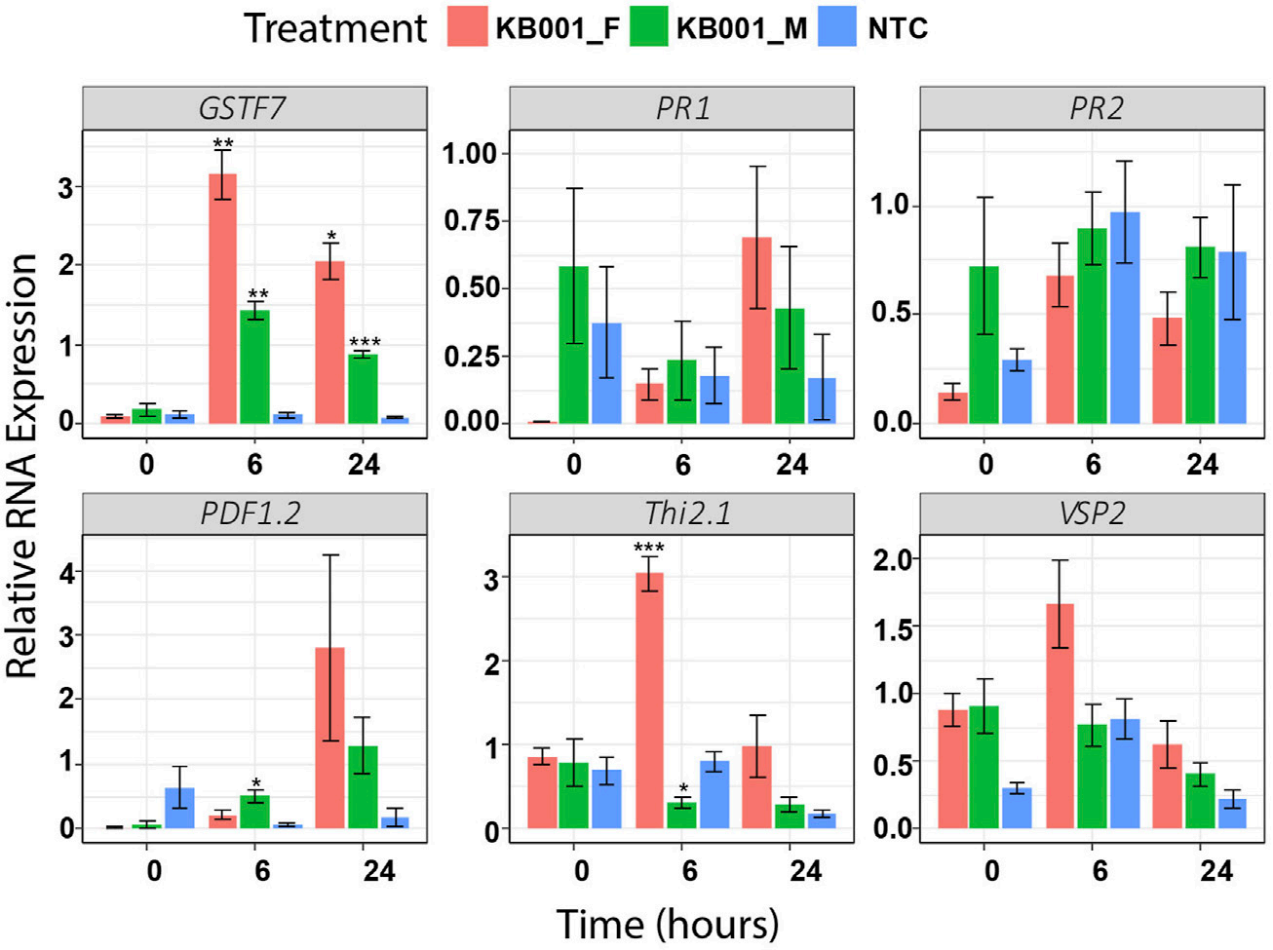
Treatment of KB001-induced stress marker expression in Arabidopsis. 10-day-old Arabidopsis seedlings were either treated with KB001 culture media (KB001_Microbe), the KB001 culture filtrate (only contains secreted compounds, KB001_Filtrate), or were not treated (NTC). RNA was extracted before treatment (time = 0), 6 and 24 h posttreatment, and gene expression was measured using qPCR. Shown is the average of four biological replicates. A *t*-test was used to determine significant differences to NTC samples. *p < 0.05; **p < 0.01; ***p < 0.001.

Based on the qPCR results of different stress markers after *S. sclerotiorum* infection, KB001 was selected as the most promising candidate for biocontrol. To distinguish whether the microbe itself or its secreted compounds would cause the enhanced defense gene expression response, we repeated the qPCR assay with Arabidopsis plants treated with either 2- to 3-week-old KB001 bacterial culture (KB001_M) or KB001 culture filtrate (KB001_F) that does not contain the bacteria. As shown in Figure 4, the KB001 filtrate treatment enhanced gene expression more than the bacterial treatment. In particular, GSTF7, Thi2.1, and VSP2 showed high responses. KB001_M treatment still showed higher expression of most genes than mock control plants. In particular, GSTF7, at both time points, and PDF1.2, at 24 h (Figure 4). The differences in response could be due to different time requirements of microbe vs. filtrate treatment and 6 or 24 h is not sufficient time for KB001 microbes to induce significantly higher gene expression in Arabidopsis. The microbial treatments may require time to colonize the plant and grow and produce specialized metabolites. The filtrate treatments already contain a range of specialized metabolites which can take effect in a shorter time frame.

Overall, these results indicate that secreted compound(s) can modify plant defense signaling faster or possibly more efficiently than the microbial treatment. It further confirms that KB001 is a good candidate for biocontrol and was therefore selected for the following *in planta* disease assays.

### Treatment of KB001 Protects Arabidopsis Plants From *Sclerotinia* and *Rhizoctonia* Infection

**FIGURE 5:**
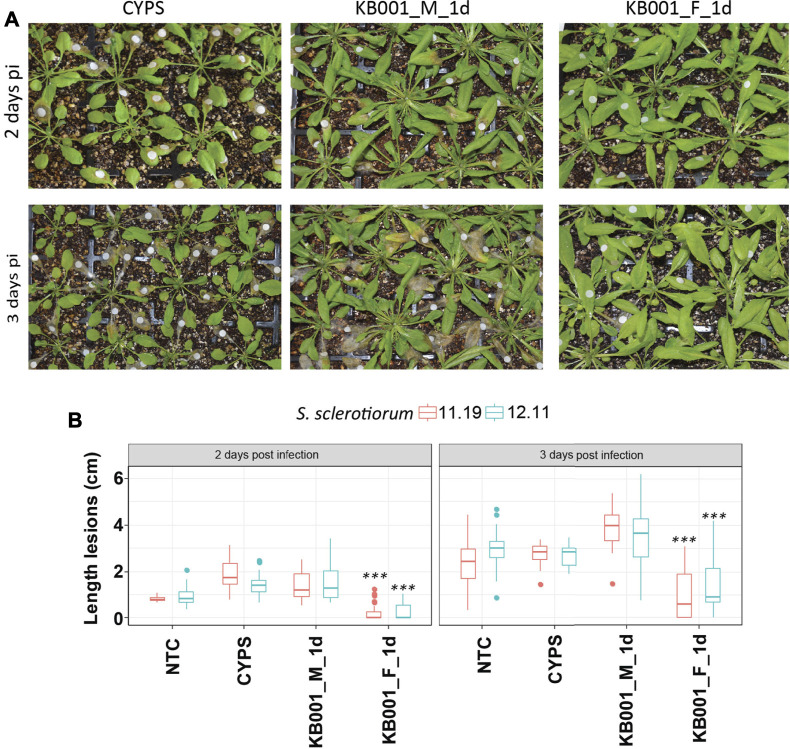
Actinobacteria filtrate improves plant fitness against *Sclerotinia* infection. Four-week-old Arabidopsis plants were treated with either KB001 microbe culture (KB001_M) or the KB001 filtrate (KB001_F) 1 day (1 day) prior to infection with *Sclerotinia*. A nontreated control (NTC) and CYPS treatment were used as references. Severity of symptoms was determined 2 and 3 days post-infection. **(A)** Plants were inoculated with *Sclerotinia sclerotiorum* isolate SS 12.11. White round Whatman paper discs show the location of inoculation. **(B)** Length of developed necrotic lesions infected with *S. sclerotiorum* strains SS 12.11 or 11.19 was measured using ImageJ software. Representative photographs are shown in 5A and Supplementary Figure S3 and were used for the analysis. Shown is the average of 20 plants per treatment. Plants were infected with two strains of *Sclerotinia* (SS 12.11 and 11.19). A *t*-test was used to determine significant differences to CYPS samples. ***p < 0.001.

To test if the Actinobacteria strain KB001 or its culture filtrate was able to improve plant fitness against pathogen infection *in planta*, Arabidopsis plants were either sprayed with 2- to 3-week-old KB001_M or KB001_F 24 h prior to infection and then subsequently inoculated with *S. sclerotiorum*. A mock treatment (CYPS media) was also included. The severity of infection was observed two and 3 days post-infection. Lesion development progressed from the white Whatman paper discs that were used to deliver the *Sclerotinia* mycelium (Figure 5A). Plants that were treated with the KB001 filtrate 24 h prior to infection showed significantly less lesion development than plants treated with CYPS media or the KB001 bacterial culture (Figure 5, Supplementary Figure S3). This indicates that the secreted compounds alone rather than the living microbe can provide protection against *Sclerotinia* during the early stage of infection. The disease assay was repeated with another *Sclerotinia* strain, 11.19, known to be highly virulent on canola (Denton-Giles et al., 2018). Infection with the 11.19 isolate produced slightly more severe symptoms (Supplementary Figure S3), but plants treated with the KB001 filtrate still showed significantly less lesion development and necrotic tissues (Figure 5B). Interestingly, plants treated with the KB001 microbial culture 24 h prior to infection showed more severe disease outbreak than control plants, and this was most pronounced at 3 days after *Sclerotinia* infection, although not at a level of statistical significance compared to the control treatment.

**FIGURE 6:**
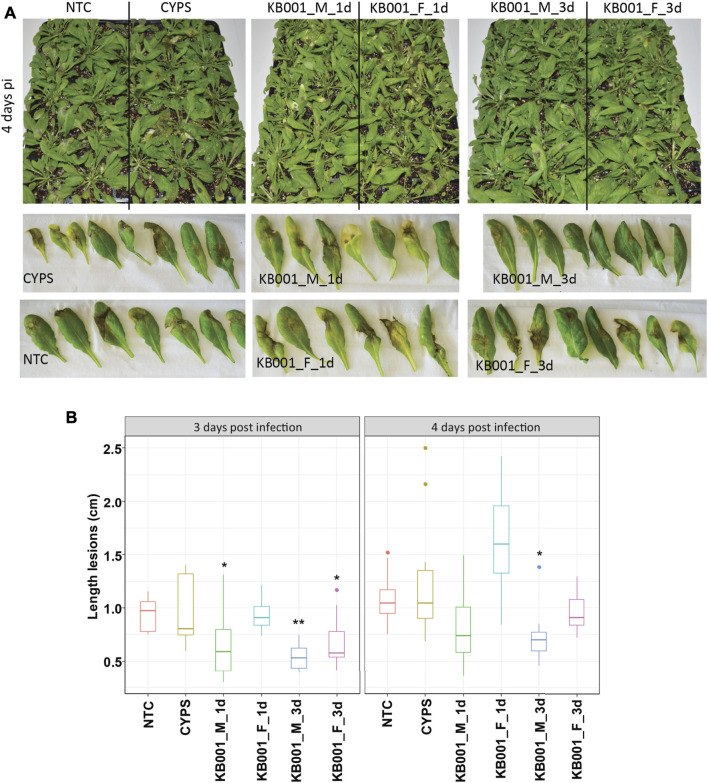
Treatment of KB001 microbe culture improved resistance to *Rhizoctonia solani* infection. 4-week-old Arabidopsis plants were treated with the KB001 microbe or culture filtrate 24 h and 3 days prior to infection with *R. solani* AG-2 ZG5. Nontreated (NTC) and CYPS-treated plants were included as references. Severity of symptoms was determined by measuring necrotic lesions 3 and 4 days post-infection. **(A)** Representative leaves inoculated with *R. solani* 4 days post-infection. **(B)** ImageJ analysis of developed lesions in infected Arabidopsis plants. Shown is the average of 12 plants. A *t*-test was used to determine significant differences to CYPS samples. *p < 0.05, **p < 0.01, ***p < 0.001.

To further investigate the ability of KB001 to protect plants from pathogens, two additional pathogens were tested. Arabidopsis plants were treated with the KB001 culture or filtrate as described before, followed by infection of *Rhizoctonia solani* (a fungal necrotroph) or *Pseudomonas syringae* (a bacterial hemibiotroph). In this assay, in addition to the 24-h KB001 culture or filtrate treatment before pathogen inoculation, we also included a 3-day pretreatment to allow additional time for the KB001 microbes to colonize the Arabidopsis plants. Indeed, treatment of KB001 microbes 3 days prior to infection slowed down disease outbreak in Arabidopsis when challenged by *Rhizoctonia*, as shown in Figure 6 for infected leaves and in Supplementary Figure S4 for the whole plant. Necrotic lesions were less severe in these plants than in the 24-h KB001 microbe and filtrate samples and significantly less developed than the CYPS control (Figure 6B). This is in contrast to the results we recorded for *Sclerotinia* infection, where the filtrate rather than the microbes increased resistance. We therefore also repeated the KB001 microbial treatment 3 days prior to infection for *Sclerotinia* infection. As observed previously, the KB001 filtrate treatment provided strong protection; however, interestingly, no increase in protection was observed with the KB001 microbial treatment (Supplementary Figure S3). This indicates that different pathways/defense responses are activated in the plant depending on both treatment and pathogen.

In case of *Pseudomonas*, no visual protection could be observed in any of the treatments (Supplementary Figure S5). Treated plants showed the same degree of symptoms as the CYPS control plants. This indicates that KB001 might act predominantly against fungal pathogens or pathogens whose lifestyles are predominately necrotrophic.

To determine if the disease protection effects were contributed by KB001-derived antifungal compounds, *in vitro* plate assays were performed between KB001 and *S. sclerotiorum* and KB001 and *R. solani* (Supplementary Figure S6). Zones of growth inhibition were observed against both *Sclerotinia* and *Rhizoctonia* when compared against their growth on plates in the absence of KB001. This confirmed that KB001 indeed has the potential to secrete antifungal compounds that act against these two fungal pathogens. This result suggests that the differences between the KB001 microbial and filtrate treatments *in planta* are due to a combination of plant defenses and antifungal compounds.

Overall, these results demonstrate that compounds produced by the Actinobacteria strain KB001 improved plant fitness and can be used as biocontrol treatments for necrotrophic fungal pathogens like *Sclerotinia* and *Rhizoctonia*. It further indicates that there is variation in the responses and/or mechanisms between different plant/Actinobacteria and pathogen combinations.

### RNAseq Analyses Reveal High Expression of Stress and Pathogen Defense Related Genes in KB001-Treated Plants

**FIGURE 7:**
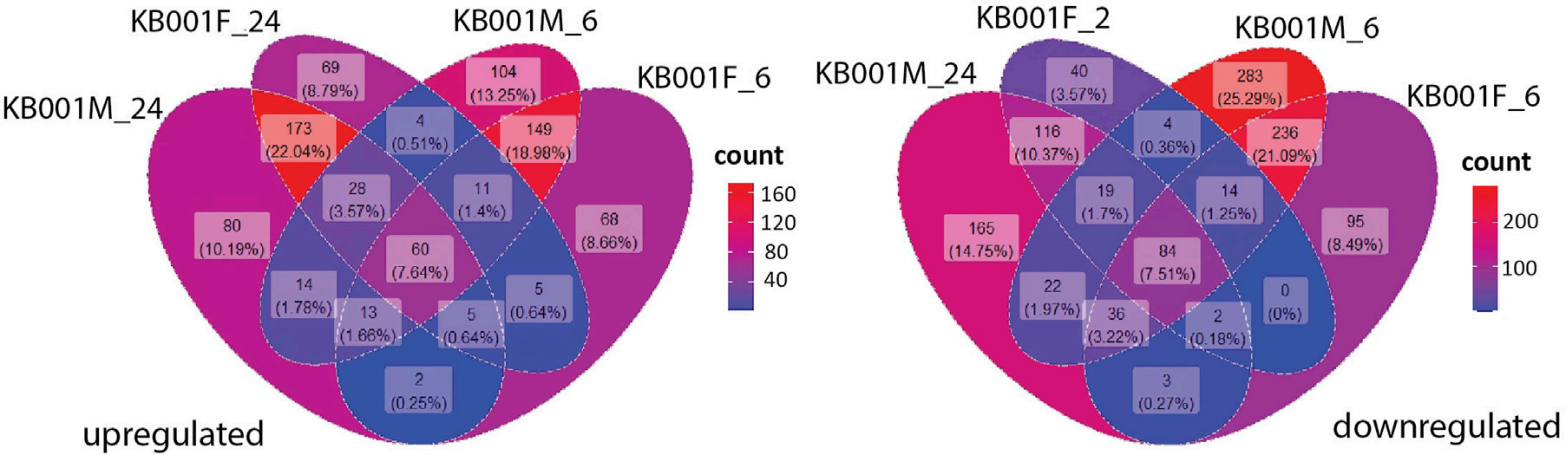
Comparison of genes up- and downregulated in the KB001 treatment groups at different time points. Pools of Arabidopsis genes expressed after the KB001 treatment groups KB001_M (microbial culture) and KB001_F (culture filtrate) 6 h (KB001M_6 and KB001F_6) and 24 h posttreatment (KB001M_24 and KB001F_24). The number (count) of genes expressed in a certain group is given in a gradient color scale.

**FIGURE 8:**
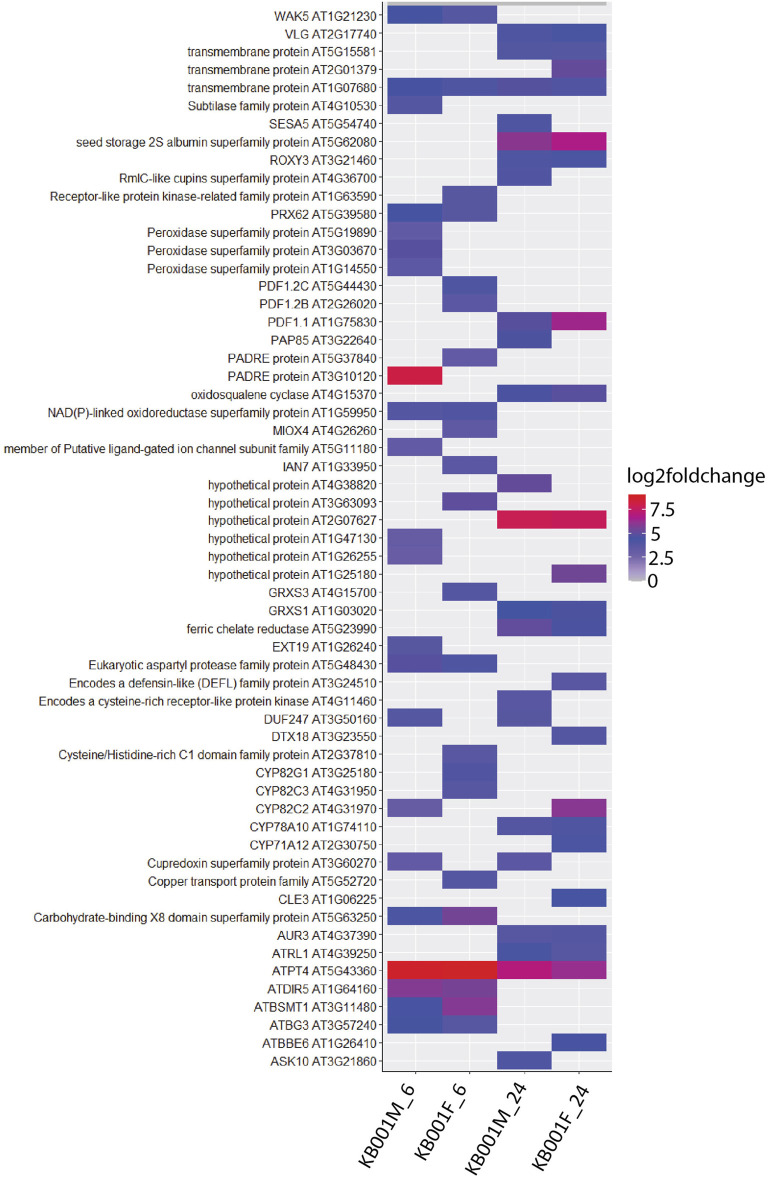
Heat map visualisation of Arabidopsis genes upregulated after KB001 microbial (KB001_M) and KB001 culture filtrate (KB001_F) treatment. Shown are the most upregulated genes in Arabidopsis after treatment with KB001 compared to the nontreated control (NTC). Four biological replicates for each treatment were used. Expression of genes is given in log2 fold change to the NTC. Description of these genes can be found in Supplementary Table S5.

Global transcriptome analysis *via* RNAseq was performed on Arabidopsis seedlings treated with either 2- to 3-week-old KB001_F or KB001_M. The aim was to identify genes that are expressed in the KB001-treated plants and are involved in the increased resistance to fungal pathogens observed in these plants. Arabidopsis seedlings grown in 24-well MS plates were treated with KB001_F or KB001_M and harvested 6 and 24 h posttreatment for RNA extraction and sequencing. Pairwise comparisons between treated plants and the nontreated control (NTC) were calculated at each time point, and up- and downregulated genes based on log2 fold change were identified (Supplementary Table S4). A description of the most differentially expressed genes in KB001 treatment groups compared to the NTC based on log2 fold change can be found in Supplementary Table S5. Genes showing a log2 fold change greater than 1 or less than −1 and a *p*-value < 0.05 (adjusted using the Benjamini–Hochberg correction) were considered for analysis. 60 genes were highly expressed in all treatment samples at both time points (Figure 7). Overall, the microbe treatment induced more unique genes only occurring in that treatment group with 104 genes at the time point 6 h and 80 at the time point 24 h. Both the filtrate and the microbial culture treatments showed substantial overlap of highly expressed genes with 149 genes at the time point 6 h and 173 at the time point 24 h, including GSTF7 (At1g02920), which was induced by 4.2-fold and 6-fold in the KB001_M and KB001_F samples, respectively, at 24 h (Supplementary Table S4). PR2 (AT3G57260), which was previously identified in the qPCR assays, was also identified by RNAseq with a 5.5-fold increase in the KB001_F sample at 6 h (Supplementary Table S4). Overlap in expression patterns was also shown for downregulated genes in the KB001-treated plants (Figure 7). The most downregulated genes were those encoding seed storage proteins and genes involved in cell wall modification. At 6 h, seed storage proteins (At4g28520 and At5g44120) were found to be downregulated in both treatments by 5–11 fold (Supplementary Table S4). Genes encoding glutamate dehydrogenase (At1g06120 and At3g03910) were downregulated by about 3.5 fold in both treatments at 24 h (Supplementary Table S4). To further illustrate overlap and differentiation of highly upregulated genes in culture vs. filtrate groups based on log2 fold change, a heat map of the most upregulated genes in KB001-treated plants compared to the NTC was designed for both time points (Figure 8, Supplementary Table S5). Expression for genes encoding peroxidases, chitinase proteins, and detoxification proteins were highly expressed at 6 h (Figure 8, Supplementary Table S5). Auxin- and gibberellic acid–associated genes were amongst the top genes expressed after 24 h (Figure 8, Supplementary Table S5). At both time points, a phosphate transporter (At5g43360) was the most upregulated gene (8–9 fold), and all samples expressed a gene encoding a transmembrane protein (At1g07680) upregulated by 4–5 fold.

To determine the type of metabolic pathways KB001 treatment induced *in planta*, we selected all genes with a log2 fold greater than 1 or smaller than −1 (*p*-value < 0.05). Their expression was visualized at the time point 24 h in relation to biotic stress response pathways using MapMan software (Supplementary Figure S7). We found that both treatment groups induced a range of genes involved in SA and JA hormone signaling but also many auxin- and ethylene-related genes (Supplementary Figure S7). In addition, several pathogenesis-related (*PR*) genes, transcription factors and other signaling molecules, and genes involved in redox control (peroxidases and GSTs) showed high expression. Amongst them are phosphate transporters and peroxidase proteins, which were highly expressed, compared to the NTC (Supplementary Table S4). In addition, several members of plant defensin proteins (PDFs), previously identified *via* qPCR, were also highly expressed in KB001-treated plants. As mentioned before, genes involved in cell wall regulation were found to be lowly expressed.

Taken together, these results indicate that the effect on gene expression of the bacterial filtrate alone is very similar to that observed with the microbial culture treatment and confirmed that the *Streptomyces* strain KB001 induced a range of redox control and plant defense-related genes. This could explain the enhanced resistance to the necrotrophic fungal pathogens *Sclerotinia* and *Rhizoctonia* which aim to cause ROS damage and host cell death.

## Discussion

By cloning a GSTF7:luc promoter:luciferase reporter construct into Arabidopsis plants and selecting for GSTF7 response in real time, we developed a suitable screening platform to identify Actinobacteria strains with potential for biocontrol. Multiple strains can be tested simultaneously, allowing for high-throughput analysis and candidate selection from large collections of isolates. Over 40 isolates were initially tested using the GSTF7:luc platform. Of those 40 isolates, 12 strains that showed the most promising GSTF7 response were selected for this study. Amongst the 12 strains, KB001 showed high GSTF7:luc responses in the screen. This was consistent with elevated endogenous GSTF7 expression and expression of stress-related genes after incubation of Actinobacteria with Arabidopsis seedlings and particularly following *Sclerotinia* infection. The KB001 isolate was identified as a *Streptomyces* (O'Sullivan et al., 2021) and consistently induced high expression of stress marker genes and, furthermore, increased resistance in Arabidopsis plants against *Sclerotinia* and *Rhizoctonia* infection but not against the bacterium *Pseudomonas. Pseudomonas syringae* is also a hemibiotrophic pathogen in that it spends some of its lifecycle feeding off living plant tissues and, in later stages, off dead tissues (Xin et al., 2018). While *S. sclerotiorum* is thought to also have a biotrophic stage, this is brief or spatially restricted (Liang and Rollins 2018). This indicates a specific bioactivity against necrotrophic fungi rather than bacteria, and the implications of defense pathways activated under these pathogen lifestyles should be considered in deploying KB001 in biocontrol. However, more bacterial pathogens would also need to be tested.

Previously, we reported another *A. thaliana* GST, GSTF8, as a marker gene for early stress responses specific to roots (Gleason et al., 2011; Thatcher et al., 2015). Within this current study, we used GSTF7 as a marker sensitive to H_2_O_2_ responses that seemed to be expressed in the whole plant, demonstrated in the bioluminescence increase and GSTF7 expression after addition of exogenous H_2_O_2_ to GSTF7:luc plants. GSTs play a physiological role during infection caused by pathogen attack (Gullner et al., 2018). For example, in a proteomic study comparing resistant to susceptible canola cultivars against the necrotrophic fungal pathogen *S. sclerotiorum*, upregulation of GSTs was observed in the resistant cultivar (Zhao et al., 2007; Zhao et al., 2009; Garg et al., 2013; Wei et al., 2016; Seifbarghi et al., 2017). This demonstrates the association of GSTs, including GSTF7, in defense mechanisms during plant–pathogen interactions.

RNAseq analysis of Arabidopsis plants treated with the KB001 microbial and culture filtrate further confirmed the influence of KB001 in plant stress and defense pathways. A series of stress markers including GSTF7, pathogen-related (PR) genes, ROS, and JA-related genes were highly expressed in both KB001 treatment groups. This is in alignment with previous studies of Arabidopsis mutants with increased expression of these signaling pathways and increased resistance to necrotrophic fungal pathogens (van Schie and Takken 2014). For example, the CPL1 gene encoding a carboxyl terminal domain phosphatase-like1 and a pathogen susceptibility gene or positive regulators of JA-mediated defense ERF5 and ERF6 induced similar gene expression. Arabidopsis cpl1 mutants and 35s:ERF5 or 35s:ERF6 overexpression lines showed higher resistance to necrotrophic fungal pathogens but not the bacterium *Pseudomonas syringae* (Moffat et al., 2012; Thatcher et al., 2018). Indeed, genes involved in biotic stress and ROS signaling, including GSTF7, and peroxidases were highly upregulated in the cpl1 mutant and E*RF* overexpression lines (Moffat et al., 2012; Thatcher et al., 2018), similar to the RNAseq profile identified in this study. Genes encoding the phosphate transporter were the most upregulated genes across all samples and time points. Previous studies showed that phosphate transporter PHT4.1 is involved in SA regulation in Arabidopsis and could thereby initiate immune responses (Wang et al., 2014). Studies in *Brassica napus* demonstrated that expression levels of many phosphate transporters were upregulated by a range of hormones, including auxin and ABA (Yang et al., 2020). This aligns with our findings of increased auxin signaling in KB001-treated plants. Glutamate dehydrogenase genes were among the most downregulated genes. Plant glutamate metabolism (GM) is important for amino acid metabolism but also has crucial roles in plant defense against pathogens (Seifi et al., 2013). During pathogen infection, the plant GM is important to maintain cell viability or induce cell death (Seifi et al., 2013). Within this study, we infected plants with necrotrophs so that downregulation of GM-related genes by KB001 could prevent cell death and infection by necrotrophic fungi.

In addition, other signaling pathways including auxin, SA, and ethylene were also induced in KB001-treated plants. This indicates that *Streptomyces* strain KB001 can manipulate multiple stress response pathways, whether directly or indirectly. Different degrees of protection were observed between the KB001_F and KB001_M treatments in Arabidopsis. The filtrate application showed higher protection against *Sclerotinia*, whereas the microbe treatment demonstrated stronger protection against *R. solani*. These results indicate a different mode of action depending on the type of application and the pathogen present. It will be important to identify which kind of application (*Streptomyces* microbes or filtrate) will provide the highest protection in the field, depending on the type of crop and the target pathogen.

Previous studies also demonstrated that inoculation of Arabidopsis with endophytic bacteria can induce systemic acquired resistance (SAR) and JA/ethylene-dependent gene expression (Conn et al., 2008). For example, endophyte-treated plants showed higher expression of defense genes once infected with a pathogen, where resistance was shown to the bacterial pathogen *Erwinia carotovora subsp. carotovora* and the fungal pathogen *Fusarium oxysporum* (Conn et al., 2008).

MapMan visualization showed that auxin signaling genes were also highly induced after 24 h in KB001-treated plants. A recent study identified 3-octanone as a *Streptomyces* compound that alters auxin/cytokinin homeostasis, resulting in the promotion of root growth (Dotson et al., 2020). Auxin signaling is known to play an important role in plant growth promotion (Zamioudis et al., 2013; Spaepen et al., 2014). Addition of exogenous auxin to Arabidopsis cell cultures showed protection from programmed cell death (PCD) (Awwad et al., 2019), a program induced by necrotrophic pathogens to increase disease development.

Recently, a *Streptomyces* strain, AgN23, was identified using a similar approach to our *GSTF7:luc* screen but using a destructive bioassay by fusing the marker gene PR1 to the *Escherichia coli* beta-glucuronidase (*GUS*) reporter gene (*PR1:GUS*) (Vergnes et al., 2019). AgN23 activated a large array of defense responses after foliar application of Arabidopsis (Vergnes et al., 2019). Vergnes et al*.* showed that AgN23 colonized the plant surface and provided protection against foliar fungal pathogens like *Alternaria brassicicola*. RNAseq analyses revealed that thousands of plant genes were activated after growing Arabidopsis hydroponically in AgN23 media, leading to production of elicitors and SA-dependent responses (Vergnes et al., 2019). Amongst the identified genes that were also confirmed by qPCR were PR5, WRKY70, Chitinase, and several others (Supplementary Table S6). These were also highly expressed in the KB001-treated plants in our study. Like KB001 studied here, AgN23 is a *Streptomyces* strain, and so an overlap in its effect on Arabidopsis gene regulation is not surprising. GSTF7 was also identified as highly induced in the *Streptomyces* AgN23 study. This further supports our use of GSTF7 as a suitable Arabidopsis marker gene for the screening of beneficial microbial strains in a non-destructive assay carried out in real time, for the potential to activate or manipulate plant defense.

Exactly how Actinobacteria induce a stress response or provide protection against fungal pathogens remains mostly unknown, but it is known that they secrete phytohormones, extracellular enzymes, and antifungal compounds (Chater et al., 2010). Some Actinobacteria are also able to induce systemic resistance by inducing plant defense responses prior to direct interactions with pathogens (Köhlet al., 2019). Our results indicate that a combination of direct antifungals produced by Actinobacteria together with the induction of plant defense genes in plants could provide increased protection, but at this stage, we cannot determine the relative importance of antifungal compounds versus induction of plant defense genes for the observed protection. While the KB001 microbial (KB001_M) and filtrate (KB001_F) treatments resulted in an overlap in differentially expressed Arabidopsis genes, the treatments also modified the expression of a significant proportion of nonoverlapping gene sets. The signaling interplay and interaction between the microbe and the plant would result in the production of metabolites and enzymes not produced under axenic growth conditions. Actinobacteria can also differ greatly in their lifestyles, and environmental factors like temperature, pH, and nutrient supply strongly influence their specialized metabolite production (Jones et al., 2017). This might explain why we observed such a diverse response of strains during the *GSTF7:luc* screen, as growing conditions might not have been optimal for all strains, or some strains might not influence the GSTF7 response in plants. We successfully demonstrated that KB001 is a strong candidate for biocontrol, but the next step is to translate the findings into the field and demonstrate protection from fungal diseases in crop plants like canola.

In conclusion, the GSTF7:luc reporter system described here can successfully be used as a screening platform for beneficial bacteria that interact with plants. High-throughput analysis allows for large numbers of bacterial isolates to be tested simultaneously. In this study, we identified *Streptomyces* strain KB001 as a strong candidate that showed increased resistance in Arabidopsis against the necrotrophic fungal pathogens *S. sclerotiorum* and *R. solani*. RNAseq and qPCR analysis confirmed its involvement in plant defense response and plant hormone signaling. This study builds a strong base for KB001 to be further investigated as a biocontrol option for crop plants.

## Data Availability

The data presented in the study are deposited in the (http://www.ncbi.nlm.nih.gov/bioproject/752867) repository, accession number (PRJNA752867), and in the (https://www.ncbi.nlm.nih.gov/nuccore/MZ669887.1/) repository, accession number (MZ669887).

## Supplementary Material

Supplementary Table S1 List of 12 Actinobacteria strains used in this study.

Supplementary Table S2 Primer sequences used for qPCR analysis. Primer sequences used in qPCR.

Supplementary Table S3 qPCR program.

Supplementary Table S4 Differentially expressed genes in KB001-treated Arabidopsis plants. Shown are the up- and downregulated genes in KB001-treated Arabidopsis plants. Genes with a log2 fold change >1 or <−1 and a *p*-value < 0.05 were considered for analysis.

Supplementary Table S5 Top 22 upregulated genes by fold change to the NTC in KB001-treated plants. Shown are the descriptions of the 22 most upregulated genes given in log2 fold change compared to the NTC in KB001-treated Arabidopsis plants.

Supplementary Table S6 Comparison of RNAseq results with Vergnes et al.’s findings. Listed are genes found in both RNAseq studies. Log2 fold change from this study and upregulation and downregulation of genes from the Verges publication are included.
