# Supplementary figures and images for: A Plant Stress-Responsive Bioreporter Coupled With Transcriptomic Analysis Allows Rapid Screening for Biocontrols of Necrotrophic Fungal Pathogens
Source: Front Mol Biosci. 2021 Sep 3;8:708530. doi: 10.3389/fmolb.2021.708530 (PMC8446517; doi:10.3389/fmolb.2021.708530)

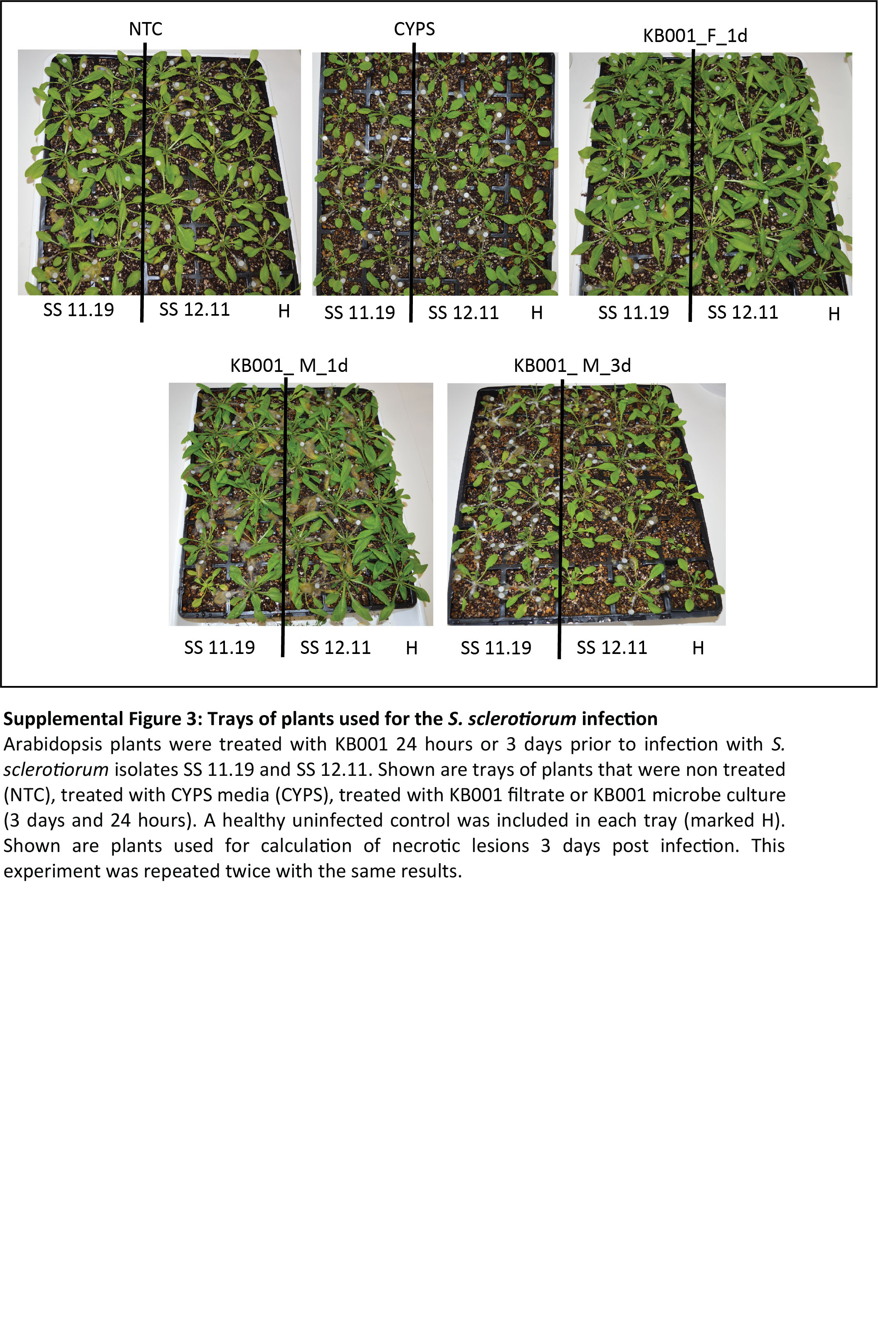

Supplement: Supplementary file 1 [file image3.jpeg]

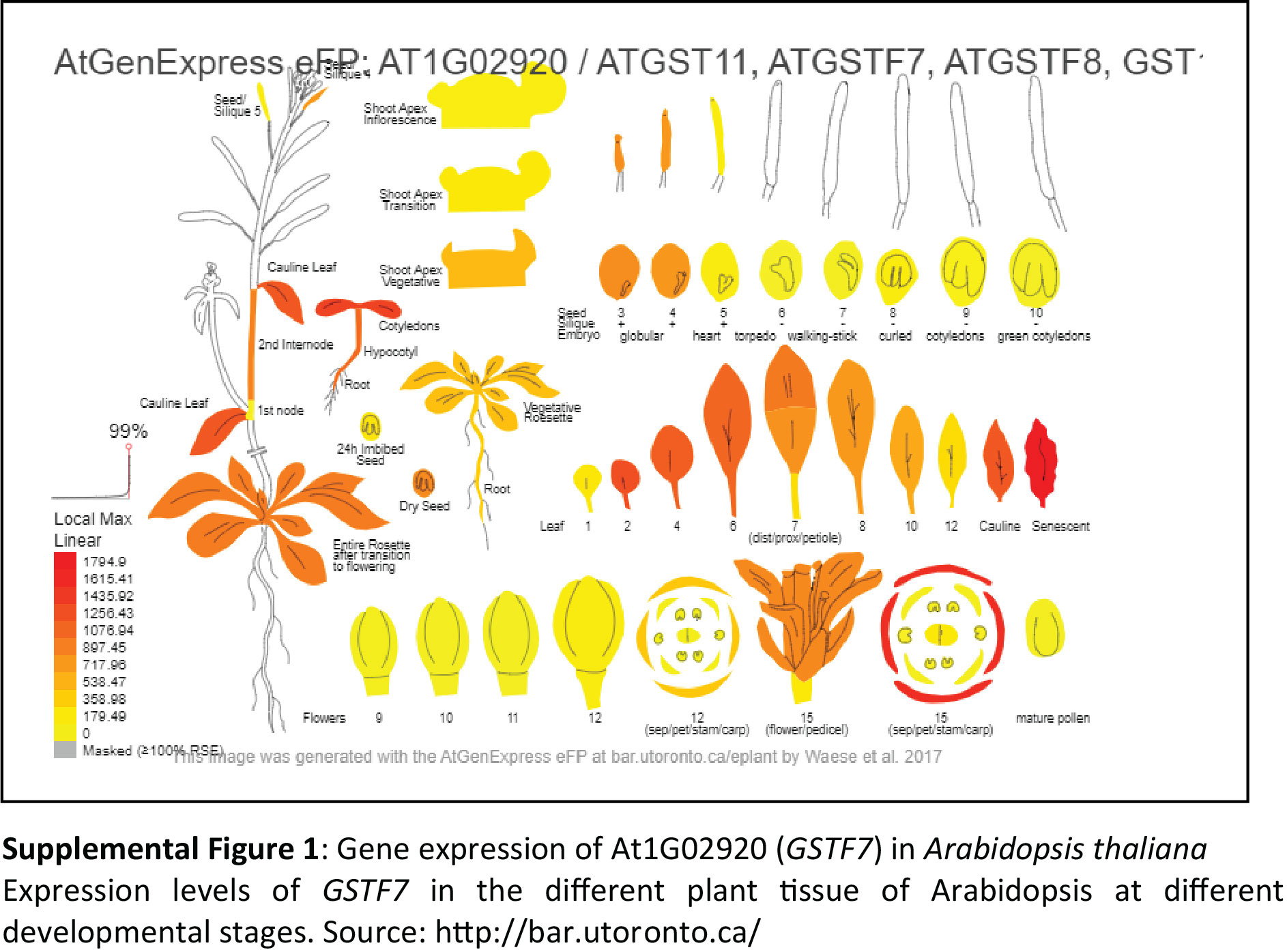

Supplement: Supplementary file 2 [file image1.jpeg]

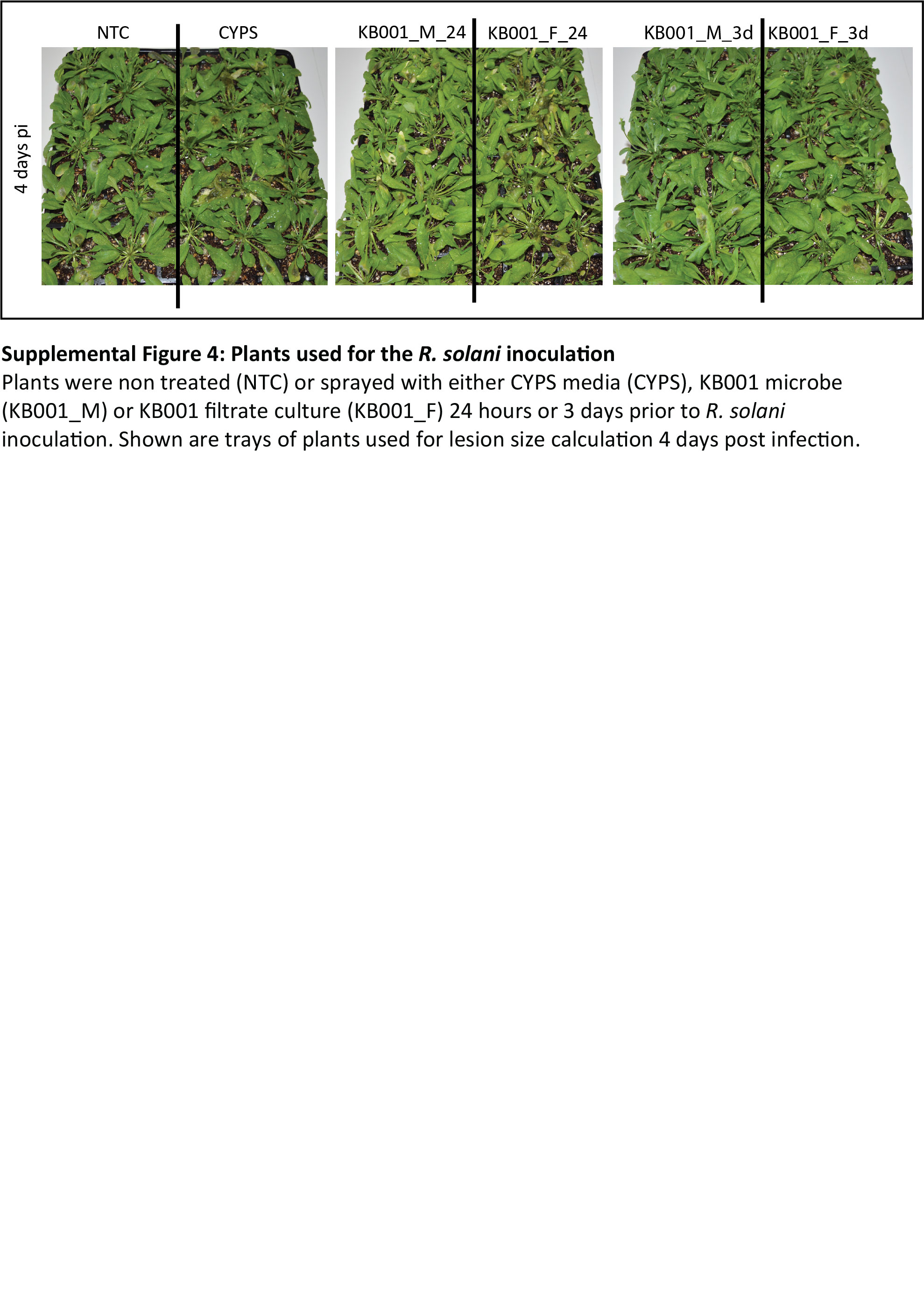

Supplement: Supplementary file 3 [file image4.jpeg]

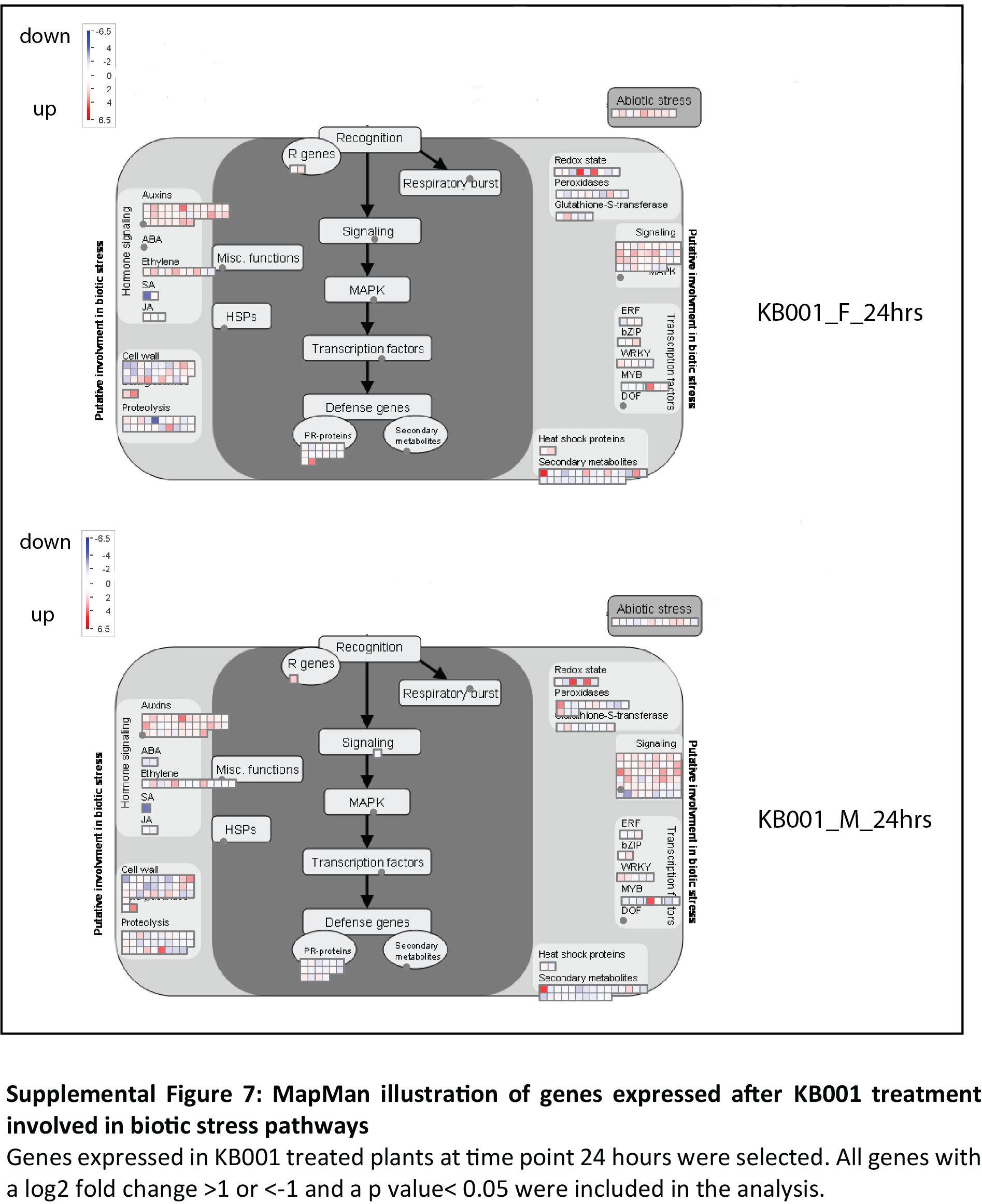

Supplement: Supplementary file 4 [file image7.jpeg]

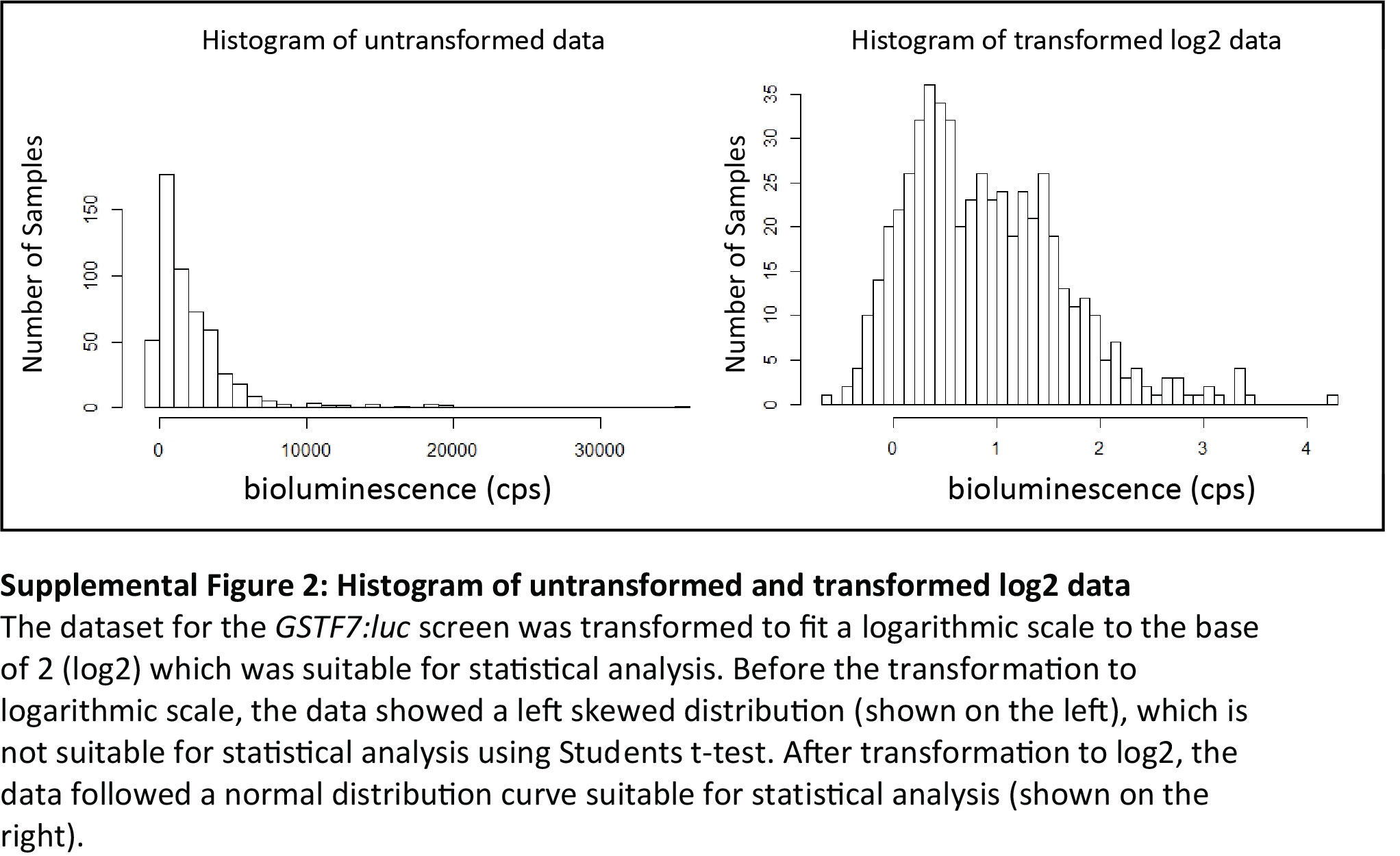

Supplement: Supplementary file 5 [file image2.jpeg]

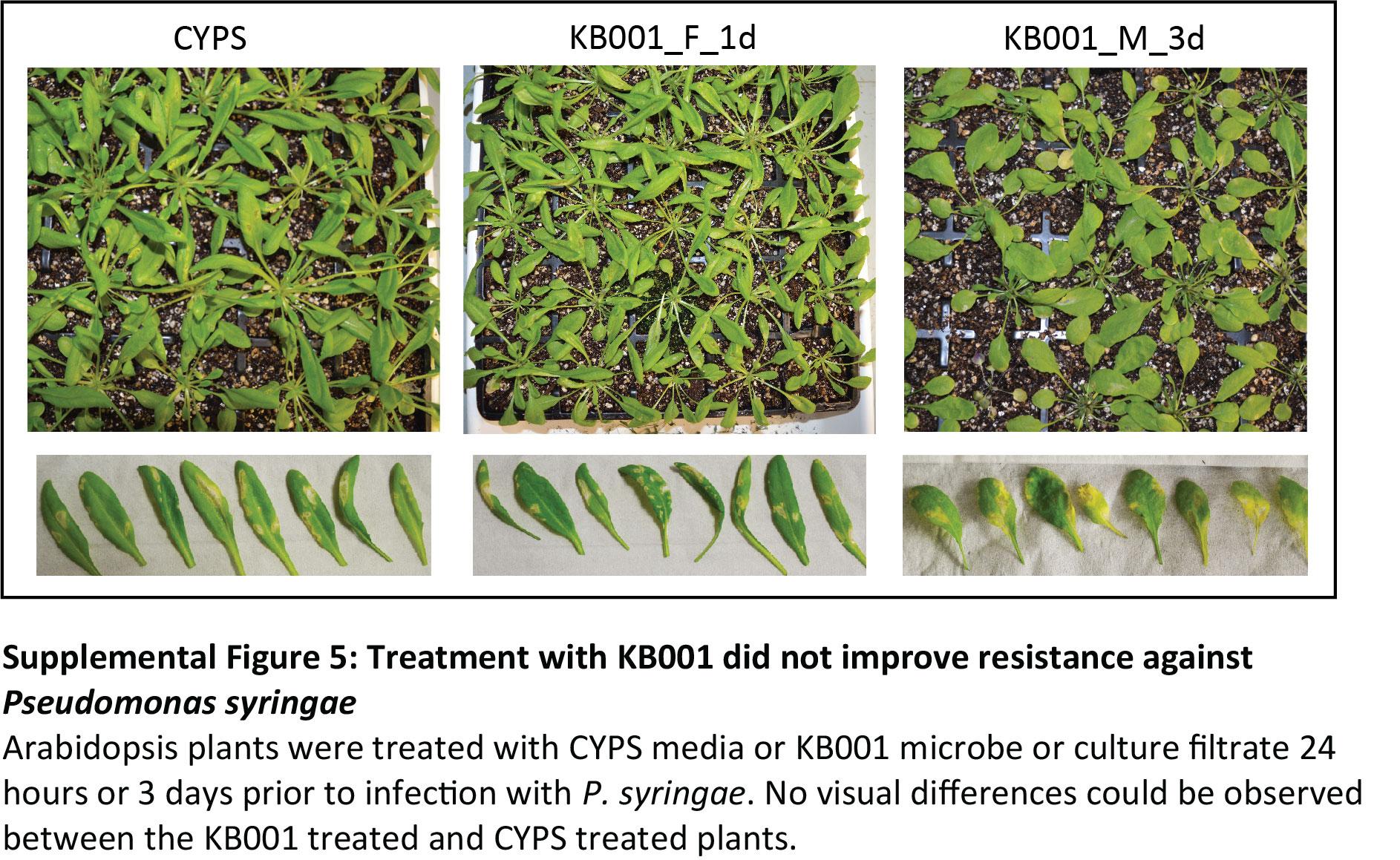

Supplement: Supplementary file 6 [file image5.jpeg]

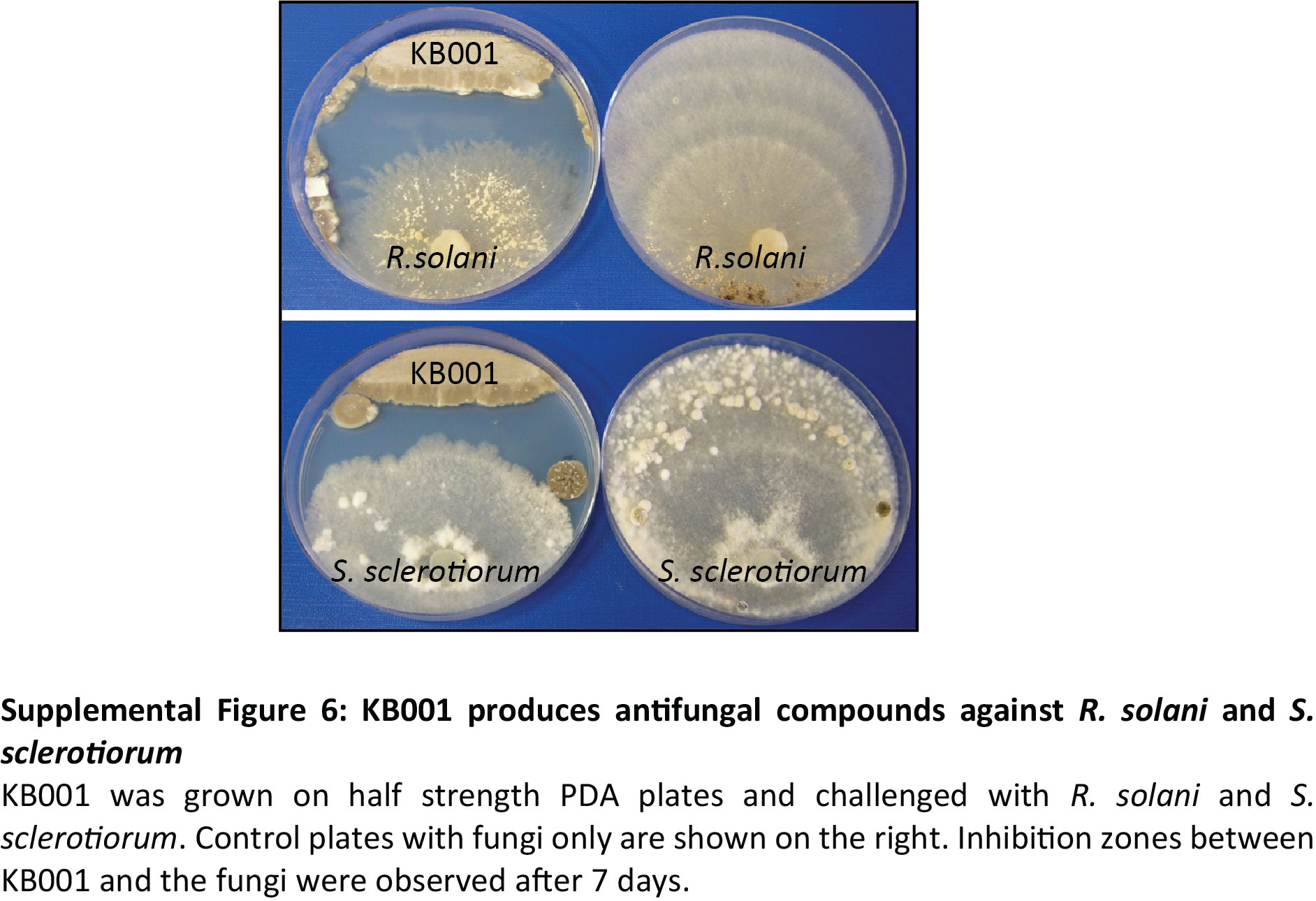

Supplement: Supplementary file 10 [file image6.jpeg]
