# Supplementary material for: A Plant Stress-Responsive Bioreporter Coupled With Transcriptomic Analysis Allows Rapid Screening for Biocontrols of Necrotrophic Fungal Pathogens
Source: Front Mol Biosci. 2021 Sep 3;8:708530. doi: 10.3389/fmolb.2021.708530 (PMC8446517; doi:10.3389/fmolb.2021.708530)
Supplement: Supplementary file 9 [file datasheet1.docx]

**Supplemental Table 1: List of 12 Actinobacteria strains used in this study**

| **Name** | **Identification** | **Reference** |
| --- | --- | --- |
| 111ww2 | *Streptomyces* | Roper et al., 2004 |
| MH12 | *Microbispora* | Unpublished |
| MH178 | *Microbispora* | O’Sullivan et al., 2021 |
| MH191 | *Streptomyces* | O’Sullivan et al., 2021 |
| MH193 | NA |  |
| MH20 | NA |  |
| MH204 | *Microbispora* | Unpublished |
| MH214 | *Streptomyces* | Unpublished |
| MH33 | *Micromonospora* | Unpublished |
| MH65 | Microbispora | Unpublished |
| KB001 | *Streptomyces* | Unpublished |
| 9a | *Streptomyces* | Roper et al., 2004 |

**Supplemental Table 2: Primer sequences used for qPCR analysis**

Primer sequences used in qPCR.

| **Primer name** | **Primer sequence** |
| --- | --- |
| PR1-F | AAGAGGCAACTGCAGACTCA |
| PR1-R | TCTCGCTAACCCACATGTTC |
| PR2-F | AGCTTAGCCTCACCACCAAT |
| PR2-R | CCCGTAGCATACTCCGATTT |
| GSTF7-F | CCACCTTGCTTTAAGAACAAAGTC |
| GSTF7-R | TTGGAGCCAAGGGAGACAAGTTGG |
| PDF1.2-F | TTTGCTGCTTTCGACGCAC |
| PDF1.2-R | CGCAAACCCCTGACCATG |
| Thi2.1 | CTCAGCTGATGCTACCAATGAGC |
| Thi2.1 | GCTCCATTCACAATTTCACTTGC |

**Supplemental Table 3: qPCR program**

| 1 | 95 °C | 2:30 min |
| --- | --- | --- |
| 2 | 95 °C | 0:15 min |
| 3 | 60 °C | 0:30 min |
| 4 | 72 °C | 0:30 min |
| 5 | Steps 2 to 4 repeated for 39 cycles |  |
| 6 | 70 °C | 0:30 min |
| 7 | 70 °C | 0:15 min + increase of 0.5 °C/sec |
| 8 | Step 7 was repeated for 50 cycles |  |
| 9 | 15 °C | 0:30min |

| VSP2-F | CCTAAAGAACGACACCGTCA |
| --- | --- |
| VSP2-R | TCGGTCTTCTCTGTTCCGTA |
| UBC-F | GCTCTCACAATTTCCAAGGTGCTGC |
| UBC-R | AGGGCTCTTCCTTAAGGACAGTATTTGTG |
| Cyclophilin-F | TCTTCCTCTTCGGAGCCATA |
| Cyclophilin-R | AAGCTGGGAATGATTCGATG |

**Supplemental Table 4: Differentially expressed genes in KB001 treated Arabidopsis plants**

Shown are the up and down regulated genes in KB001 treated Arabidopsis plants. Genes with a log2 fold change >1 or <-1 and a p value <0.05 were considered for analysis.

**Supplemental Table 5: Top 22 up regulated genes by foldchange to NTC in KB001 treated plants**

Shown are the descriptions of the 22 most up regulated genes given in log2 foldchange compared to NTC in KB001 treated Arabidopsis plants.

**Supplemental Table 6: Comparison of RNAseq results with Vergnes et al findings**

Listed are genes found in both RNAseq studies. Log2 foldchange from this study and up and downregulation of genes from the Verges publication are included.

| **Gene** | **Name** | **Vergnes et al** | **Sample** | **log2 fc** |
| --- | --- | --- | --- | --- |
| AT1G02920 | GSTF7 | upregulated | KB001_F_24, KB001_M_24 | 2.59, 2.06 |
| At1G75040 | PR5 | upregulated | KB001_F_6 | 2.01 |
| At3G56400 | WRKY70 | upregulated | KB001_F_24 | 1.12 |
| At2G30750 | CYP71A12 | upregulated | KB001_M_24, KB001_F_24 | 3.49, 4.18 |
| At5G25260 | Flotilin2 | upregulated | KB001_M_24 | 1.54 |
| At5G46330 | FLS2 | upregulated | KB001_M_6, KB001_M_24 | 1.26, 1.04 |
| At4G35770 | SEN1 | upregulated | KB001_F_6, KB001_F_24 | 1.27, 1.25 |
| At2G43570 | Chitinase | upregulated | KB001_F_24, KB001_M_24 | 1.76, 1.46 |
| At5G43580 | Serine Protease inhibitor | upregulated | KB001_M_6, KB001_F_6, KB001_F_24 | 1.38, 1.35, 1.03 |
| At1G75830 | LCR67 | upregulated | KB001_F_24, KB001_M_24 | 6.46, 4.85 |
| At2G23170 | GH3.3 | upregulated | KB001_M_6, KB001_F_24, KB001_M_24 | 1.14, 3.17, 2.77 |
| At5G22300 | Nitrilase 4 | downregulated | KB001_F_24 | -1.48 |
| At5G01600 | Ferretin 1 | downregulated | KB001_M_24 | -1.6 |
| At5G56870 | BGAL4 | downregulated | KB001_M_24 | -1.31 |
| At2G18660 | Plant Natriuretic Peptide A | upregulated | KB001_M_6 | -1.57 |
| At5G57050 | ABA Insensitive 2 | downregulated | KB001_M_6 | -1.05 |
| At2G45220 | Pectin Methylesterase 17 | upregulated | KB001_M_24 | -1.7 |
